# Assessing brain metastasis response to immunotherapy: a pictorial review of atypical responses and intracranial adverse events

**DOI:** 10.1186/s13244-025-02125-z

**Published:** 2025-11-19

**Authors:** Gary Amseian, Francisco Aya, Camilo Pineda, Sofía González-Ortiz, Juan-Andrés Mora, Maria-Lourdes Olondo, Andres Perissinotti, Gabriela-Ailen Caballero, Iban Aldecoa, Laura Mezquita, Josep Puig, Ana Arance, Núria Bargalló, Laura Oleaga

**Affiliations:** 1 Department of Radiology, Hospital Clínic de Barcelona, Barcelona, Spain; 2 Universitat de Barcelona, Barcelona, Spain; 3 Department of Medical Oncology, Hospital Clínic de Barcelona, Barcelona, Spain; 4 Institut d’Investigacions Biomèdiques August Pi i Sunyer (IDIBAPS), Barcelona, Spain; 5 Department of Nuclear Medicine, Hospital Clínic Barcelona, Barcelona, Spain; 6 Centro de Investigación Biomédica en Red de Bioingeniería, Biomateriales y Nanomedicina (CIBER-BBN), ISCIII, Barcelona, Spain; 7 Department of Pathology, Biomedical Diagnostic Center (CDB), Hospital Clínic Barcelona, Barcelona, Spain; 8 Neurological Tissue Bank of the Biobank-IDIBAPS, Barcelona, Spain; 9 Laboratory of Translational Genomics and Targeted Therapies in Solid Tumors, IDIBAPS, Barcelona, Spain

**Keywords:** Brain metastasis, Immunotherapy, Response assessment, Pseudoprogression, Immune-related adverse events

## Abstract

**Abstract:**

Immunotherapy, particularly immune checkpoint inhibitors, plays a crucial role in the treatment of brain metastases in various primary cancers. Response assessment encompasses atypical patterns, including pseudoprogression, hyperprogression, or dissociated response, which present greater complexity than classical patterns defined by standardized response assessment criteria. Additionally, intracranial adverse events like hypophysitis or encephalitis may resemble tumor progression. Accurate evaluation and management of brain metastases during immunotherapy requires that radiologists are familiar with both classical and atypical response patterns, as well as potential intracranial adverse events. Brain MRI and advanced imaging techniques serve as essential tools for this purpose.

**Critical relevance statement:**

Assessing brain metastases response to immunotherapy accurately is fundamental for therapeutic decision-making. Radiologists must recognize classical and atypical responses and adverse events associated with immunotherapy to ensure optimal patient management.

**Key Points:**

- Immunotherapy response assessment in brain metastases is complex due to atypical patterns including pseudoprogression, hyperprogression, and dissociated responses.
- Immunotherapy-induced intracranial adverse events, such as hypophysitis and encephalitis, must be accurately identified.
- Brain MRI, complemented by advanced imaging techniques (perfusion MRI, MRS, and amino acid PET), is crucial for distinguishing these complex scenarios.

**Graphical Abstract:**

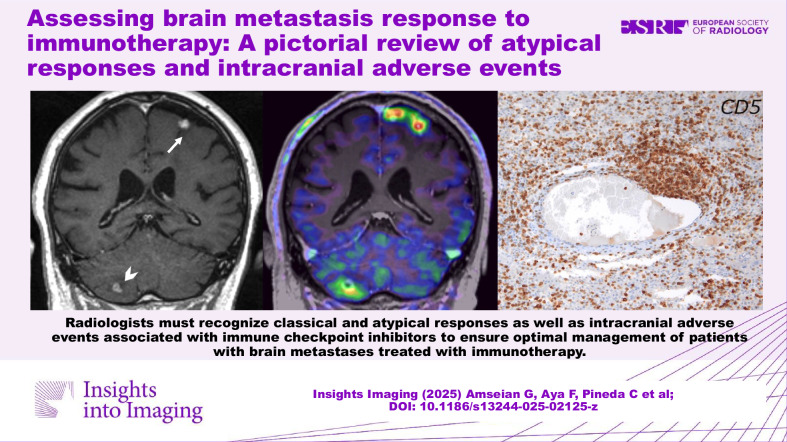

## Introduction

Brain metastases represent the most common intracranial neoplasms in adults, impacting around 20% of patients with cancer, with autopsy series indicating a true incidence that may reach 40% [1]. The frequency of brain metastases is increasing, largely due to improvements in cancer care, such as earlier detection and more effective treatments, which have prolonged patient survival and thereby increased time for metastases to develop. Although any primary tumor has the potential to metastasize to the brain, the most frequent origins are lung cancer (20–56% of patients), breast cancer (5–20%), and melanoma (7–16%) [2].

The management of brain metastases is challenging due to their resistance to conventional therapies, including cytotoxic chemotherapy. This resistance is compounded by the blood-brain barrier (BBB) and other factors such as tumor barrier heterogeneity, elevated interstitial pressure, molecular adaptations, and irregular perfusion, leading to poor efficacy and prognosis [3]. The brain has traditionally been considered “immune-privileged” due to the BBB and lack of dendritic cells and lymphatic structures. However, evidence from conditions like multiple sclerosis and brain tumors [4], along with the detection of tumor-infiltrating T lymphocytes in brain metastases [5], has challenged this perspective, showing that immune cells can access the brain under certain conditions.

Immune checkpoint inhibitors (ICIs) block proteins that are overexpressed by cancer cells, which serve to inhibit the immune system response against tumors, such as PD-1, PDL-1, or CTLA-4. This mechanism facilitates the recognition and destruction of cancer cells by T lymphocytes in the brain [6]. Examples comprise anti-PD-1 agents (Pembrolizumab, Nivolumab), anti-PDL-1 agents (Durvalumab), and anti-CTLA-4 agents (Ipilimumab). Historically, patients with brain metastases were either excluded or underrepresented in clinical trials. However, in recent years, a limited number of trials specifically designed for patients with brain metastases have emerged, providing promising data on the safety and efficacy of ICIs in this population. These studies have shown encouraging intracranial responses, particularly in asymptomatic patients not requiring corticosteroids, and have contributed to the recognition of immunotherapy as an effective therapeutic option in selected cases [7, 8]. Indeed, clinical guidelines from organizations like the European Society for Medical Oncology (ESMO) and the National Comprehensive Cancer Network (NCCN) currently incorporate immunotherapy as a first-line treatment option for brain metastases from different primary tumors, including melanoma [9, 10] and non-small cell lung cancer [11, 12].

The treatment of brain metastases is tailored to patient characteristics and combines multimodal strategies. These include local interventions such as surgery and radiotherapy, as well as systemic therapies, which encompass corticosteroids, chemotherapy, targeted therapies, and immunotherapies. Brain imaging establishes baseline patient status, facilitates treatment selection, and assesses immunotherapy response for guiding decisions regarding the continuation, adjustment, or discontinuation of treatment.

## Imaging brain metastases

In patients with brain metastases, contrast-enhanced brain MRI is the gold standard minimally invasive test to evaluate disease burden before starting immunotherapy and to monitor treatment response during follow-up [13]. NCCN guidelines stipulate that follow-up imaging should be performed every 2 or 3 months during the first year or 2 years after treatment initiation, and subsequently every 4 to 6 months indefinitely [14]. CT scanning exhibits lower sensitivity than MRI; nonetheless, it is advantageous for rapid intracerebral evaluation in acute settings and for identifying neurosurgical emergencies. It is also used for planning stereotactic radiosurgery (SRS) [15] and in conditions when MRI is contraindicated, such as in patients with certain pacemakers.

Standardizing MRI acquisition protocols is essential for achieving consistent measurements and ensuring reliable assessment of response. Table 1 shows the standardized protocol for the study of brain metastases used in the authors’ center, following the consensus recommendations for a standardized brain tumor imaging protocol for clinical trials in brain metastases proposed by Kaufmann et al in 2020 [16]. This protocol aligns with the consensus recommendations for a standardized brain tumor imaging protocol in clinical trials proposed in 2015 by Ellingson et al [17].

**Table 1:** Standardized brain tumor MRI protocol for clinical trials in brain metastases proposed by Kaufmann et al in 2020

Sequence	Notes/purpose
T1 3D (volumetric)	Baseline anatomical evaluation.
T2-FLAIR axial 2D	Detection of perilesional edema and lesions.
DWI axial	Assessment of diffusion restriction (ischemia, cellularity).
**Administration of intravenous contrast agent**	
T2 axial 2D	Performed after contrast injection but before post-contrast T1 3D sequence.
T1 3D (volumetric) post-contrast	Detection and characterization of lesion enhancement.
T1 Spin-Echo axial 2D	Optional/Additional to improve lesion conspicuity.

The sequences in the table are listed in order of acquisition. The use of 3-T MRI over 1.5-T is recommended, if possible, due to its increased sensitivity to detect small lesions. Adapted from Kaufman et al [16]

Spin-echo T1 sequence is incorporated at the end of the imaging protocol, as it provides superior tissue contrast and reduced sensitivity to magnetic field inhomogeneities, thereby improving the detection of subtle parenchymal enhancement. In addition, a T1 SPACE sequence, SPACE (Siemens), VISTA (Philips), or CUBE (GE) may be considered, as it offers high-resolution isotropic imaging and could serve as a suitable replacement for the MPR sequence. Their intrinsic black-blood contrast also suppresses vascular signal, thereby enhancing the delineation of lesions adjacent to vessels. These features make them particularly suitable for evaluating brain metastases and leptomeningeal disease, and for improving diagnostic accuracy in treatment monitoring. Adopting SE T1 and 3D isotropic T1 sequences enhances protocol robustness and optimizes lesion detection in the context of brain metastasis imaging under immunotherapy.

Contrast agents are a critical part of the imaging exam. Evidence from multicenter studies suggests that agents with high relaxivity, such as gadobenate dimeglumine, have high diagnostic efficiency, allowing for equivalent diagnostic image quality using a lower dose [18]. This allows for the reduction of total gadolinium exposure, which is especially valuable in settings where multiple follow-up scans are necessary. Therefore, the highest relaxivity agent available should be used, and it should be the same at each follow-up time point. The contrast agent used in the authors’ institution (and the one used for the acquisition of the images included in this review) is Gadoteric acid, a macrocyclic gadolinium-based contrast agent (GBCA). The standard dose used is 0.2 cc/Kg.

Throughout this review, MRI contrast-enhanced T1-weighted images will illustrate lesion assessment, unless stated otherwise. The images included in this study were acquired using magnetic field strengths of either 1.5 T or 3 T.

## Available criteria for assessing brain metastases response to immunotherapy

The RECIST guidelines [19] provide a standardized method for assessing the response of extracerebral tumors to cytotoxic chemotherapy in advanced solid cancers. The Response Assessment in Neuro-Oncology (RANO) criteria for high-grade gliomas (RANO-HGG) were concurrently developed to improve the reliability of response assessment in glioma trials [20]. The RANO working group, upon recognizing the limitations of existing criteria for assessing brain metastases, proposed new criteria known as RANO-BM [21], which integrates features of RECIST 1.1 and RANO-HGG. The criteria assess tumor burden through one-dimensional measurement of target lesions exceeding 10 mm and track tumor size changes during follow-up. Both the RANO-HGG criteria and the subsequently developed immunotherapy-specific iRANO criteria [22] have been superseded by their most recent version, RANO 2.0 [20], while the RANO-BM criteria remain in effect. The RANO-BM criteria are one of the main reference systems in clinical trials for brain metastases, and a summary of their main characteristics is provided in Table 2. In parallel, modified RECIST (mRECIST) criteria [23] have been developed to better address the specific challenges of assessing response in patients with brain metastases. Notably, mRECIST allows the inclusion of up to five intracranial lesions measuring ≥ 5 mm, in contrast to the 10 mm threshold required by standard RECIST or RANO-BM. This adaptation is particularly relevant given the earlier detection of small-volume brain metastases through high-resolution MRI. Although methodological differences exist between criteria, there is a significant level of concordance among them [24]. These criteria classify treatment response into four categories: complete response (CR) is defined as the disappearance of all lesions; partial response (PR) indicates a significant decrease in tumor burden compared to baseline, though not complete; progressive disease (PD) is characterized by a significant increase in tumor burden compared to nadir; and stable disease (SD) applies when the response does not fulfill the criteria for other categories.

**Table 2:** Summary of RANO-BM assessment criteria, which are currently one of the main reference systems in clinical trials for brain metastases

Measurable lesions	≥ 10 mm in one diameter
Target lesions	5
Complete response (CR)	Complete disappearance of all lesions. No steroid use. Clinical status stable/improved
Partial response (PR)	≥ 30% decrease in SLD compared to baseline. Stable/less steroids. Clinical status stable/improved
Progressive disease (PD)	≥ 20% increase in SLD compared to nadir, with a minimum absolute increase of 5 mm in one lesion. Significant T2 signal increase. New lesions. Clinical status worse
Stable disease (SD)	Does not meet criteria for CR, PR, or PD

*SLD* sum of longest diameters

Immunotherapy trials have questioned traditional classifications by revealing radiological changes indicative of delayed responses or inflammation induced by therapy. These findings highlight the need for specific response assessment criteria in patients receiving immunotherapy. To this end, the specific criteria for immunotherapy iRECIST [25] and iRANO [22] were developed. Their main contribution was the recognition that clinical benefits, including long-term survival and tumor regression, can occur even following initial progression or appearance of new lesions. However, novel patterns of response and progression to immunotherapy, including hyperprogression, dissociated response, and sustained response, have been reported beyond pseudoprogression. These patterns do not conform to classical patterns and are not encompassed within the specific immunotherapy criteria [26]. Recognizing atypical and complex radiological patterns is crucial for accurately assessing treatment responses.

## Pseudoprogression

Pseudoprogression (PsPD) refers to a temporary increase in lesion size and/or number on imaging during early follow-up after initiating treatment, even when patients are responding favorably to therapy. This phenomenon has been documented in other neuro-oncological scenarios, including gliomas undergoing chemoradiotherapy [27]. In brain metastases treated with immunotherapy, initial observations were made in melanoma patients receiving an anti-CTLA-4 antibody, where some individuals showed early enlargement of tumor lesions, followed by a decrease or stabilization of tumor burden in later evaluations [28]. PsPD has also been reported in patients receiving anti-PD-1 and anti-PD-L1 therapies [29].

**Fig. 1:**
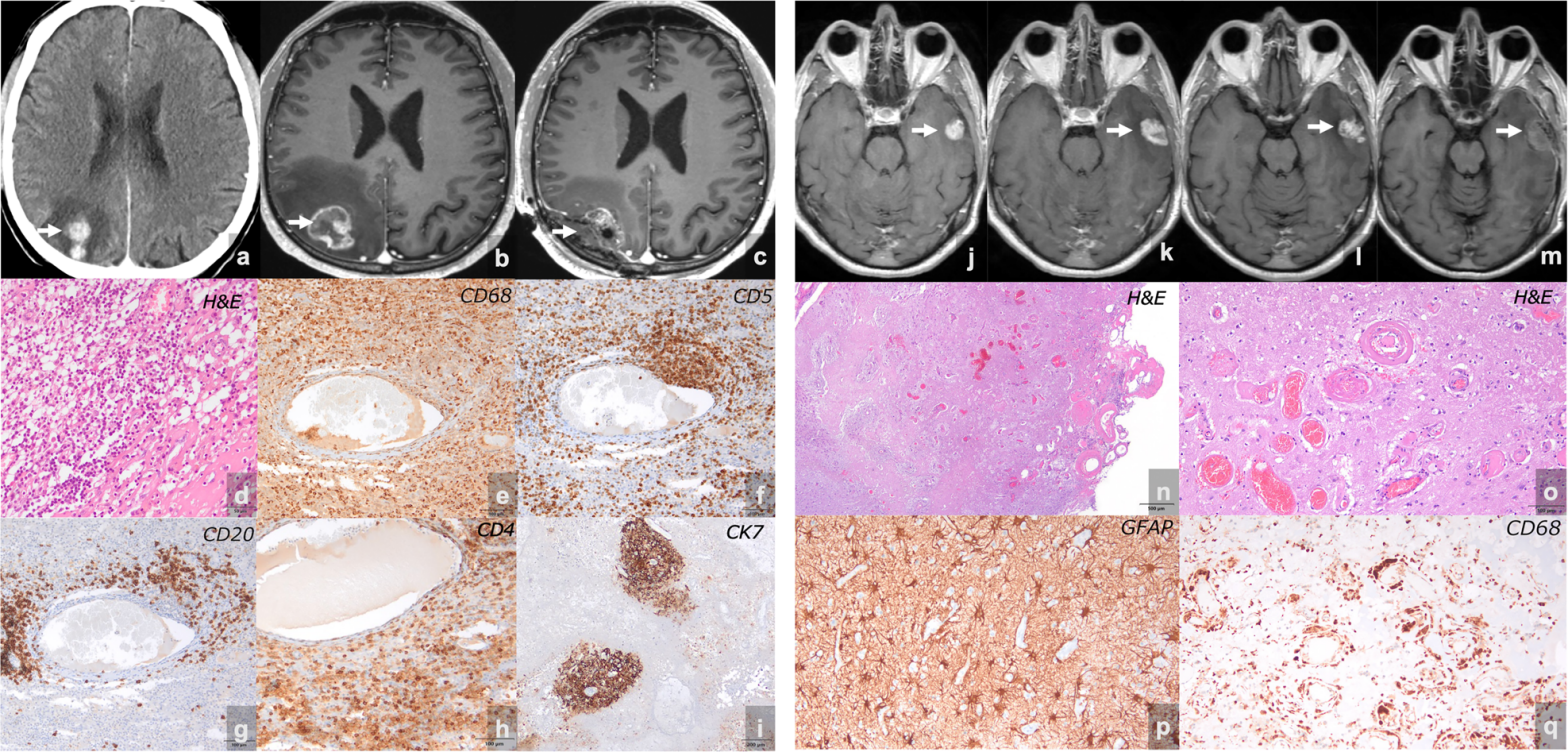
Radiopathological correlation of pseudoprogression (**a**–**i**) and radiation necrosis (**j**–**q**). Pseudoprogression (**a**–**i**) in a patient with PD-L1 > 50% NSCLC and brain metastasis treated with Pembrolizumab without concomitant radiotherapy. Baseline CT showed a parieto-occipital metastasis (**a**). An MRI 38 days later revealed an increase in both lesion size and edema (**b**). Following surgical resection (**c**), histopathological analysis of the resected lesion (**d**–**i**) revealed extensive necrosis (**d**) and abundant immune infiltrates of T cells, B cells, T helper cells (**e**–**h**), with scattered foci of neoplastic cells (**i**), suggesting an immune-mediated response. Radiation necrosis (**j**–**q**) in a patient with NSCLC brain metastasis treated with Pembrolizumab and SRS. A temporal lobe lesion (**j**) initially showed an increase in size on MRI 187 days post-treatment (**k**). Subsequent imaging 585 days later (**l**) showed stable enhancement. The lesion was resected (**m**), and its histopathology (**n**–**q**) revealed extensive coagulative necrosis (**n**), vascular hyalinosis (**o**), gliosis (**p**), immune cell infiltration (**q**), and no tumoral cells, confirming radiation necrosis

On MRI, PsPD is characterized by a transient increase in tumor burden and gadolinium-enhanced signal intensity, often accompanied by increased perilesional edema; these findings eventually stabilize or decrease. These changes result from inflammatory and vascular modifications from immunotherapy-induced immune activation. Histopathological findings include necrosis, hemorrhage, edema, astrogliosis, lymphocytic infiltration (CD4⁺ and CD8⁺ T cells), as well as plasmacytic and macrophage infiltrates. Additionally, there is microglial infiltration alongside areas of tumor regression and tumor replacement by connective tissue [30, 31]. The imaging features of these histopathological alterations closely resemble those of true tumor progression (Fig. 1).

The incidence of PsPD in patients with brain metastases receiving immunotherapy ranges from 1% to 10%, depending on primary tumor type [32]. There is no consensus on timing; it can occur between the first and 18th week after treatment initiation, rarely beyond 6 months. Conventional response criteria may underestimate immunotherapy benefits, potentially resulting in misguided decisions such as premature treatment discontinuation. To address this, immunotherapy-specific response criteria emphasize the necessity of time intervals before confirming progression based on size increase, provided the patient’s clinical condition remains stable. The RECIST 1.1 criteria have been adapted to iRECIST [25], which advises a minimum interval of 4–8 weeks before confirming definitive progression. Similarly, the RANO 2.0 [20] criteria propose that if an increase in lesion burden is observed within the first 3 months after the start of treatment, a second, sequential study should be performed at least 4 weeks later before confirming progression. To confirm progression, the second study must show a significant increase in lesion burden (> 25% increase in the product of the perpendicular diameters of the lesions, or a > 40% increase in the total volume of contrast-enhancing lesions). If the second follow-up shows SD or a partial/complete response (PR/CR), the initial apparent progression is classified as pseudoprogression, and the patient should continue the treatment.

## Radiation necrosis

An important proportion of patients with brain metastases treated with immunotherapy also undergo radiotherapy, either stereotactic radiosurgery (SRS) for those with a limited number of lesions or whole brain radiotherapy (WBRT) for patients with extensive disease and multiple metastases. Cerebral radiation-induced injury encompasses a range from acute, reversible edema to late, irreversible radiation necrosis (RN). In RN, radiation induces endothelial damage, causing hypoxia, edema, and immune activation, which ultimately leads to necrosis of the affected brain tissue. RN is a delayed adverse effect of SRS, affecting 5–25% of treated patients [33]. The risk escalates with increased radiation doses, larger treatment volumes, and in patients who have also received WBRT [34]. The median duration for the development of RN post-radiotherapy is 6–11 months, with an incidence rate of roughly 15 to 17% at 12 months. Significant RN is uncommon within the initial 6 months; however, it can manifest years after treatment, with the cumulative incidence reported to rise to 25–34% at 2 years in some studies [33]. Histopathologically, RN is characterized by well-circumscribed regions of eosinophilic coagulative necrosis, which are surrounded by normocellular brain parenchyma. This tissue exhibits edema, mild gliosis, and vascular hyalinization [35, 36] (Fig. 1).

RN manifests with symptoms, such as headache, nausea, and somnolence, which can be indistinguishable from those associated with tumor progression. Radiologically, both RN and progressive disease exhibit increased T2 signal intensity, which indicates edema and alterations in the blood-brain barrier, leading to enhanced contrast uptake [37] (Fig. 1). In high-grade gliomas, distinct MRI contrast enhancement patterns have been proposed to differentiate PD (focal solid nodules; solid uniform enhancement with distinct margins) from RN (hazy mesh-like diffuse; feathery indistinct rim enhancement) [38], though applicability to brain metastases is uncertain. Similar to PsPD, the features of RN in brain metastases may be indistinguishable from PD when assessed using standard imaging protocols. Nevertheless, an accurate diagnosis is crucial for proper therapeutic decision-making.

Advanced imaging techniques to distinguish pseudoprogression and radiation necrosis from progressive disease.

In patients with brain metastases undergoing immunotherapy, standard MRI lacks the reliability to differentiate PsPD from true disease progression, often requiring up to 6 months of follow-up to confirm progression. In patients who have also received radiotherapy, similar changes occurring beyond 6 months may be attributed to RN. In certain clinical scenarios where timely therapeutic decisions are critical, additional imaging modalities, including advanced MRI techniques such as perfusion, spectroscopy or radiolabeled amino acid positron emission tomography (PET), are essential for distinguishing between these entities within a constrained timeframe.

## Apparent diffusion coefficient (ADC)

**Fig. 2:**
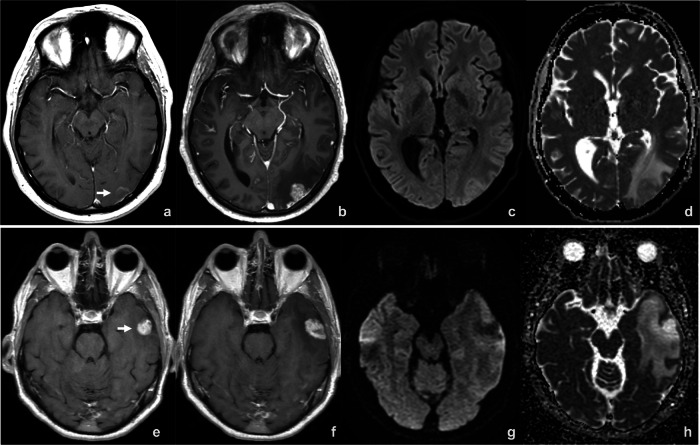
Diffusion imaging in two patients with brain metastases treated with immunotherapy. Patient 1 (**a**–**d**): A melanoma patient treated with Nivolumab–Ipilimumab and WBRT presented with a left occipital metastasis (arrow in **a**). Follow-up at 104 days (**b**) showed lesion enlargement with increased signal on DWI (**c**) and a mean ADC of 0.938 × 10⁻³ mm²/s (**d**), consistent with progressive disease. Patient 2 (**e**–**h**): An NSCLC patient treated with Pembrolizumab and SRS had a left temporal metastasis (arrow in **e**). At 187 days (**f**), the lesion enlarged without DWI signal increase (**g**) and showed a mean ADC of 2.861 × 10⁻³ mm²/s (**h**). The lesion remained stable and was ultimately resected, with histopathology confirming radionecrosis

Although a standard MRI technique, the Apparent Diffusion Coefficient (ADC) is particularly noteworthy. Derived from diffusion-weighted imaging (DWI), ADC provides a quantitative measure of water molecule mobility within tissues. Typically, a low ADC indicates high cellularity (solid lesions), while a high ADC suggests lower cellularity, necrosis, or edema due to freer water movement. ADC can serve as an early, sensitive indicator of treatment efficacy for brain metastases (Fig. 2). A study on lung cancer metastases revealed that ADC values increased significantly with each treatment cycle in responders. In contrast, values in non-responders remained stable before decreasing [39]. Specifically in immunotherapy, one study found that a brain metastasis patient treated with an anti-CTLA-4 agent had a significantly higher ADC than untreated patients. Histologically, these high-ADC areas correlated with lower vessel density, higher macrophage and T-cell infiltration, increased necrosis, and decreased cell proliferation [40]. Combined with other advanced MRI techniques, ADC helps differentiate tumor progression from post-treatment changes [41].

## Perfusion magnetic resonance imaging

**Fig. 3:**
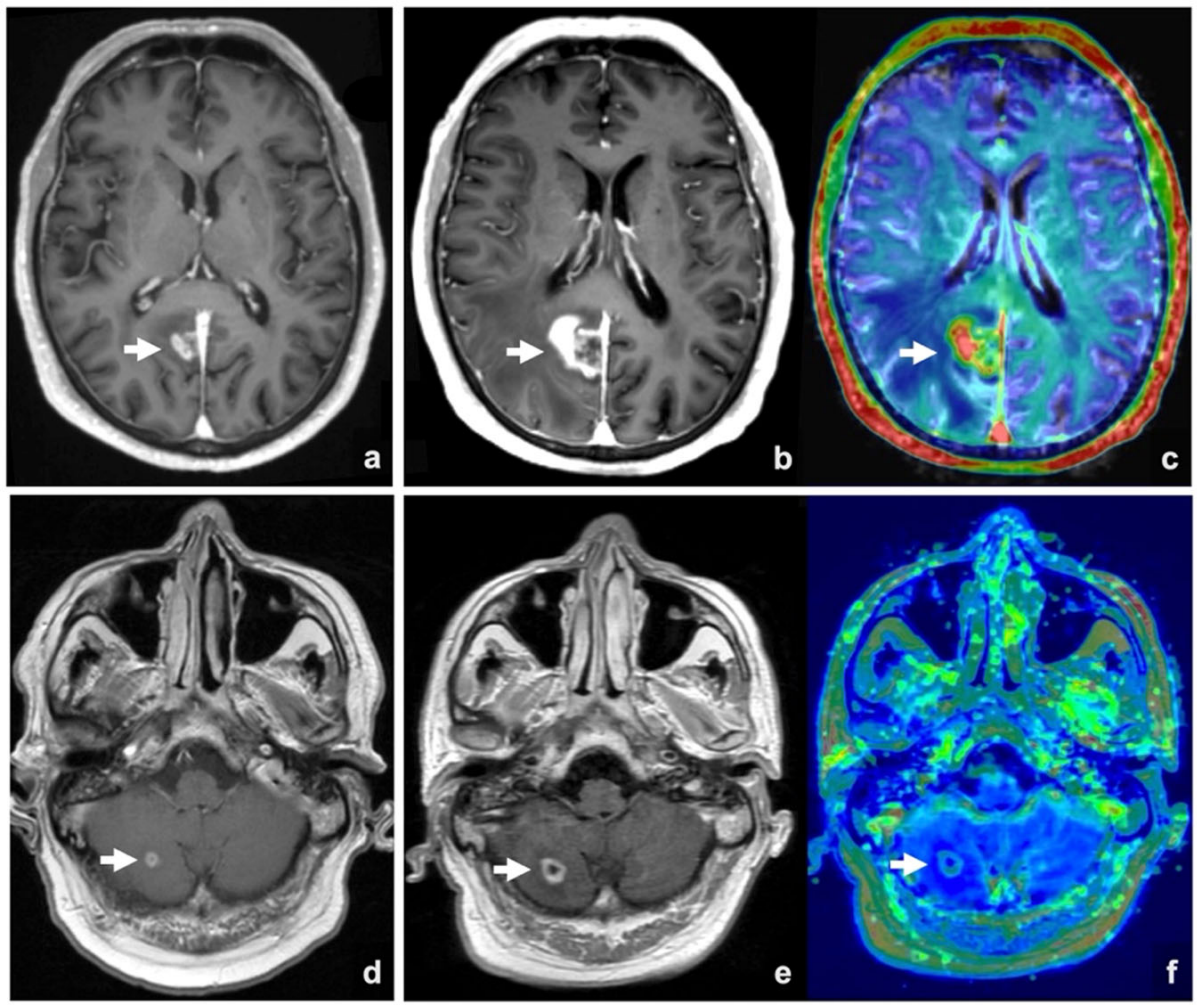
Perfusion MRI in two different patients with brain metastases from NSCLC treated with Pembrolizumab. The first patient was treated without concomitant radiotherapy, while the second also received SRS. In the first patient (**a**–**c**), contrast-enhanced T1w brain MRI (**a**) shows a right occipital lesion (arrow in **a**). One year later (**b**), the lesion has increased in size, and perfusion MRI (**c**) shows a pathological increase in CBV, indicating disease progression. In a second patient (**d**–**f**), brain MRI (**d**) shows a right cerebellar lesion (arrow in **d**). Five months later (**e**), the lesion has increased in size; however, perfusion MRI (**f**) shows no significant increase in CBV, findings that are consistent with post-treatment changes

Perfusion MRI quantifies vascularization and hemodynamics in brain lesions by assessing cerebral blood volume (CBV) and brain permeability. CBV represents the total volume of blood within a designated region, quantified in milliliters of blood per 100 grams of tissue, and is obtained via DSC. Permeability refers to the efficacy of molecular passage across the blood vessel wall, measured by the time-dependent leakage constant Ktrans, which is obtained via DCE. In neuro-oncology, these parameters facilitate lesion characterization, treatment response evaluation, and therapeutic decision-making. Brain neoplastic lesions typically exhibit neoangiogenesis, compromise the BBB  [42], increase perfusion, and show elevated CBV and Ktrans values. In this context, for lesions that have increased in size on follow-up brain MRI, true PD presents with hyperperfusion with elevated CBV and Ktrans values when compared to PsPD [43] (Fig. 3). However, recent studies suggest that immunotherapy-induced pseudoprogression might be characterized by increased blood perfusion compared to chemoradiotherapy-induced pseudoprogression or necrosis. This difference could be due to a more active inflammatory response caused by the activation of the immune system, with infiltration of immune cells and local inflammatory changes that increase perfusion. Further studies are needed to refine the utility of perfusion in distinguishing between different post-treatment changes [44].

Perfusion sequences such as dynamic contrast-enhanced (DCE) and dynamic susceptibility contrast (DSC) imaging require the use of contrast agents. DSC perfusion uses T2 or T2*-weighted sequences, making it prone to susceptibility artifacts caused by blood products, calcifications, or proximity to bone, which can complicate measurements and make them unreliable [45]. In contrast, DCE perfusion uses T1-weighted sequences and, although its acquisition and processing can be more complex, it is less susceptible to these artifacts, allowing for a superior quantitative evaluation of cerebral blood volume and offering better spatial resolution. Therefore, in the presence of hemorrhage or other elements that cause magnetic susceptibility, DCE is considered a more robust and reliable technique for differentiating tumor progression from post-treatment changes [46].

## Magnetic resonance spectroscopy

**Fig. 4:**
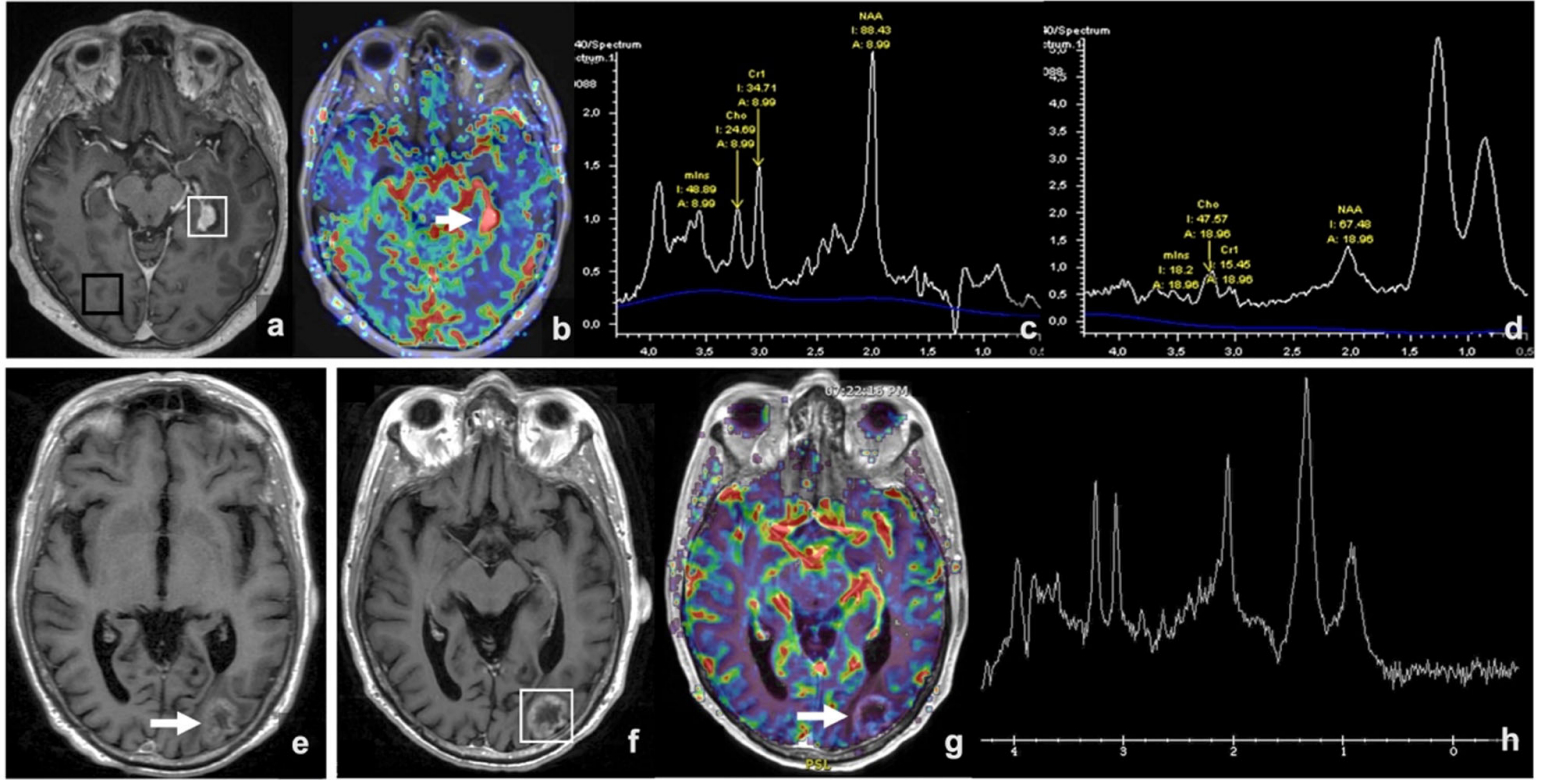
MRS in two different patients with brain metastases from renal cell carcinoma treated with Pembrolizumab. The first patient was treated without concomitant radiotherapy, while the second also received SRS. In the first patient (**a**–**d**), a new lesion was identified during follow-up (white square in **a**), showing increased CBV on perfusion imaging (arrow in **b**). Spectroscopy of the normal brain parenchyma (**c**, from the black square in **a**) revealed a typical pattern with elevated NAA and a Cho/Cr ratio below 1. MRS of the lesion (**d**, from the white square in **a**) demonstrated decreased Cho and Cr, an elevated Cho/Cr ratio (~3), marked NAA reduction, and a Lip-Lac peak, consistent with a tumoral lesion due to progressive disease. Excisional biopsy confirmed the lesion as a new metastasis. In the second patient (**e**–**h**), a known metastatic lesion (arrow in **e**) shows an increase in size on follow-up (white square in **f**) without associated CBV increase on perfusion imaging (arrow in **g**). MRS of the lesion (**h**, from the white square in **f**) revealed a mild relative increase in Cho and Cr with a Cho/Cr ratio of ~1, slight relative NAA reduction, and increased Lip-Lac, suggestive of post-treatment changes. Subsequent follow-up confirmed the stability of the lesion

Magnetic resonance spectroscopy (MRS) allows the analysis of tissue chemical composition, represented graphically with the X-axis indicating the chemical shift in parts per million (ppm) and metabolites appearing at specific positions, and the Y-axis denotes the signal intensity, which correlates to the metabolite concentration. Different molecules are depicted, which serve as markers of physiological processes and are altered in different pathophysiological contexts; the most relevant in this context are listed in Table 3. Tumor progression is marked by elevated Cho levels and reduced NAA levels. The Cho/Cr ratio tends to be higher in the progressing tumors compared to post-treatment changes. PsPD is characterized by MRS revealing decreased levels of Cho, Cr, and NAA (Fig. 4), which suggests tissue damage rather than active tumor growth. RN is additionally associated with elevated levels of Lip and Lac [47, 48]. Although the utility of MRS in evaluating treatment response in brain metastases treated with immunotherapy is promising, especially as part of a multiparametric approach, it has limitations, such as the potential overlap of spectroscopic findings and limited availability in some medical centers. Furthermore, specific clinical evidence for immunotherapy is still emerging, and its use is considered complementary in combination with other advanced imaging techniques.

**Table 3:** Main metabolites in MRS according to their chemical shifts (ppm)

Chemical shift (ppm)	Metabolite	Pathophysiologic processes
3.2	Choline (Cho)	Cellular turnover (increased in tumors and proliferative processes)
2.0	N-acetyl aspartate (NAA)	Neuronal viability (decreased in neuronal pathologies)
3.0	Creatine (Cr)	Internal control due to its relative stability
1.3	Lactate (Lac)	Anaerobic metabolism (found in necrotic tissues)
1.3	Lipids (Lip)	Cell membrane components (elevated in high cell turnover)

Adapted from Weinberg et al [47]

## Overview of MRI techniques

A recent systematic review and meta-analysis assessed the diagnostic accuracy of currently available MRI techniques for treatment response evaluation in patients with brain metastasis (not specific to immunotherapy) [49]. The sensitivities and specificities found for the different techniques can be seen in Table 4.

**Table 4:** Diagnostic accuracy of currently available MRI techniques for treatment response evaluation in patients with brain metastasis

Technique	Sensitivity	Specificity
Conventional MRI	79%	76%
DCE perfusion	74%	92%
DSC perfusion	83%	78%
Diffusion-weighted imaging	67%	79%
MRS	80%	78%
Combined techniques	84%	88%

Adapted from Teunissen et al [49]

Although specific data on immunotherapy are still needed, it seems that a multiparametric correlation of MRI techniques offers the highest diagnostic accuracy for differentiating tumor progression from treatment-induced changes. A lesion that increases in size or enhancement after treatment is suspicious. If this lesion shows low ADC, high rCBV, and an elevated choline peak, it is highly suggestive of tumor progression. If, on the other hand, it shows high ADC, low rCBV, and an elevated lipids/lactate peak, it is more likely to be pseudoprogression or a treatment effect. Despite the high sensitivity and specificity of the multiparametric approach, the different techniques may not be available, or the analysis results may not be conclusive. In centers where the technique is available, it is possible to resort to a nuclear imaging technique, Radiolabeled Amino Acids PET.

## Radiolabeled amino acids PET

**Fig. 5:**
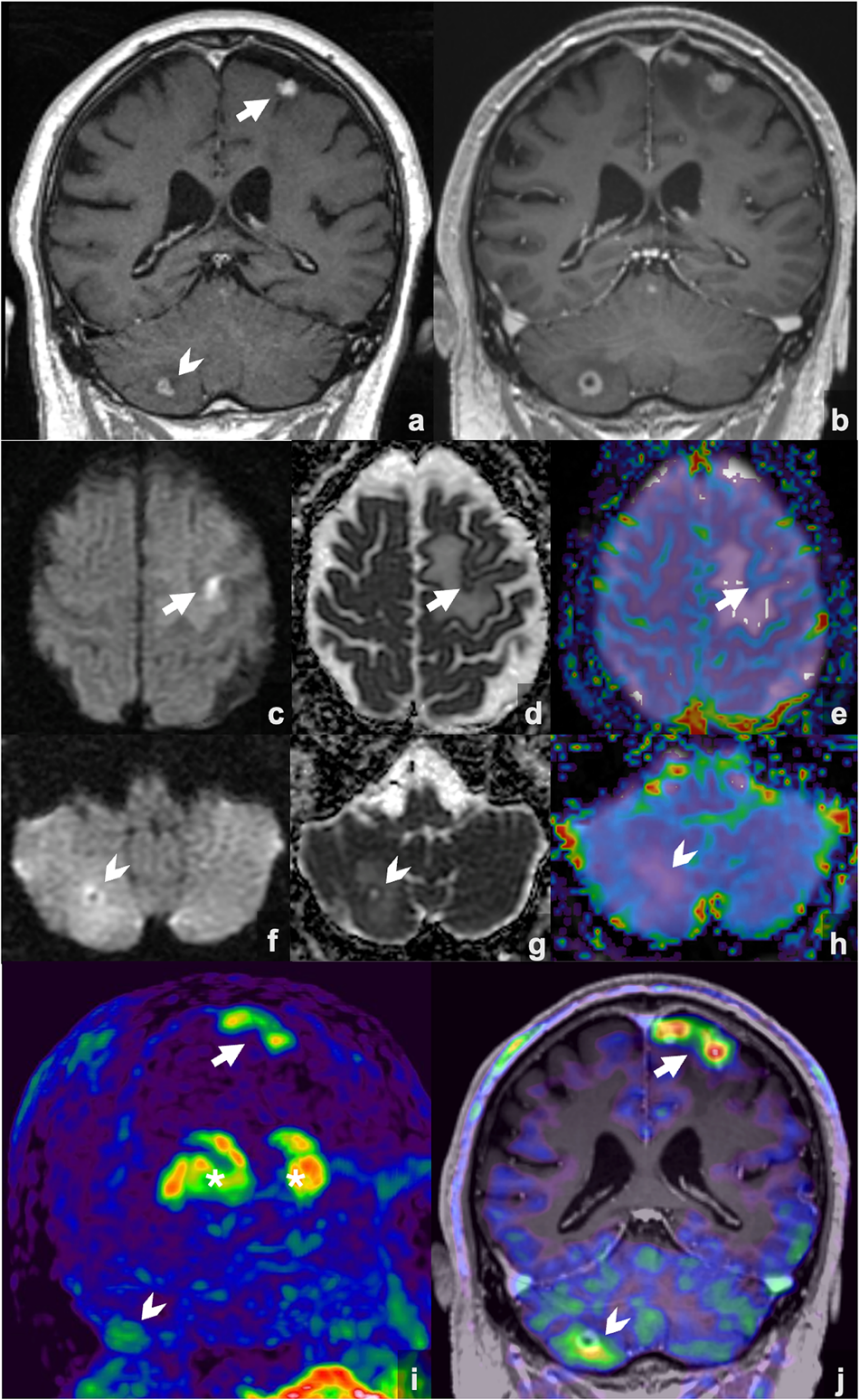
Brain MRI and ^18^F-FDOPA PET in NSCLC brain metastases after Pembrolizumab and SRS. A left frontal cortico-subcortical lesion (arrow) and a right cerebellar hemisphere lesion (arrowhead) are shown (**a**). One year post-treatment, MRI (**b**) shows enlargement of both lesions and a new nodularity adjacent to the frontal lesion. Diffusion and perfusion imaging were inconclusive for the frontal lesion (DWI **c**, ADC **d**, rCBV **e**). The cerebellar lesion showed peripheral increased signal on the DWI (**f**) with decreased ADC (**g**) but no rCBV increase (**h**). ^18^F-FDOPA PET (3D reconstruction **i**, fused MRI-PET **j**) demonstrated two foci of uptake in the frontal lesion (arrow), consistent with active disease, while the cerebellar lesion showed only mild uptake (arrowhead), suggesting post-treatment changes. Physiologic ^18^F-FDOPA uptake is noted in the basal ganglia (*)

PET imaging with radiolabeled amino acids (^11^C-MET, ^18^F-FET, and ^18^F-FDOPA) serves as a crucial tool for assessing treatment response in neuro-oncology, as tumor cells show high uptake due to overexpression of amino acid transporters [50]. As opposed to the ^18^F-FDG tracer, the lower physiological aminoacidic uptake in normal brain tissue enhances the tumor-to-background ratio, facilitating improved visualization of tumor margins. Effective therapies typically lead to decreased tracer uptake, while increased tracer uptake during treatment suggests therapeutic failure (Fig. 5).

This molecular imaging technique can be useful to distinguish tumor recurrence from treatment-related changes, including RN or PsPD [51]. Specific studies in the context of immunotherapy have shown that amino acid PET can achieve high diagnostic accuracy. In a study with a mixed cohort of patients with brain metastases treated with immunotherapy and targeted therapies, an accuracy of 85% was observed for differentiating tumor relapse from treatment-induced changes, with a specificity of 94% and a sensitivity of 70%, using well-defined tumor-to-brain ratio thresholds [52]. In 2024, the RANO group published the PET-based response assessment criteria for diffuse gliomas (PET RANO 1.0) to standardize the use of amino acid PET imaging in both clinical trials and practice [53]. The aforementioned criteria may also be relevant for assessing brain metastases until specific guidelines for this patient group are established. Other applications include defining the optimal biopsy site and delineating tumor extent for surgical and radiotherapy planning.

**Fig. 6:**
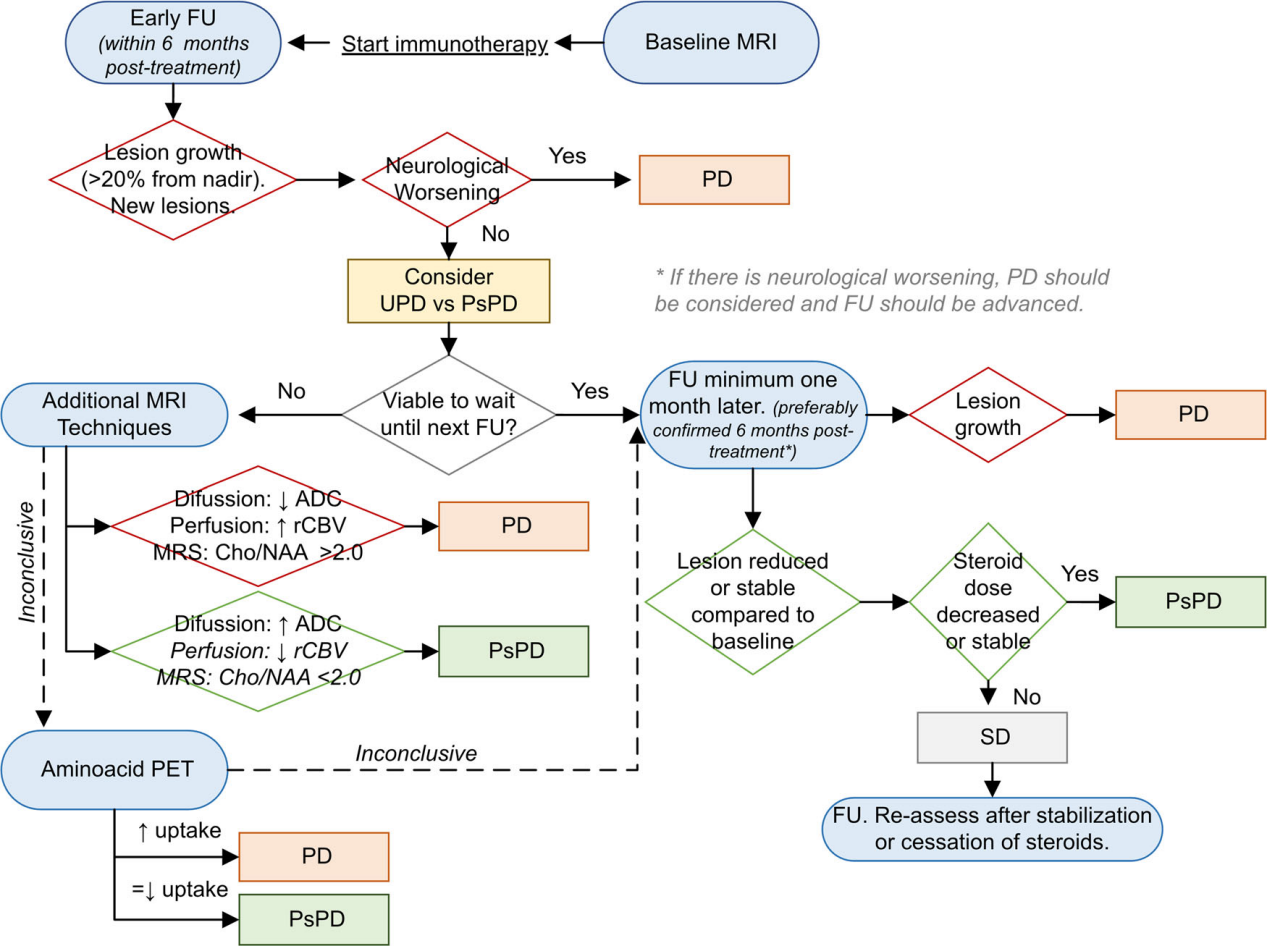
Proposed algorithm to differentiate between tumor progression and pseudoprogression during the first 6 months of follow-up after initiating immunotherapy. This approach starts with a standard brain MRI protocol, assessing the size and number of contrast-enhancing lesions. In cases of ambiguous results with increased lesion size, where waiting for the next follow-up is not feasible, assessment of additional MRI techniques such as ADC, perfusion, or spectroscopy is recommended. When brain MRI techniques are inconclusive, amino acid PET can be used. In patients who have received radiotherapy and present with increased lesion size more than 6 months after the administration of radiotherapy, a similar, chronologically adapted algorithm can be used to differentiate between true progression and radiation necrosis. FU, follow-up; MRS, magnetic resonance spectroscopy; PD, progressive disease; PsPD, pseudoprogression; SD, stable disease; UPD, unconfirmed progressive disease

While the choice of diagnostic imaging techniques varies depending on each center’s availability and expertise, an algorithm is proposed in Fig. 6 for monitoring patients with brain metastases treated with immunotherapy to differentiate between tumor progression and post-treatment changes. This approach starts with a standard brain MRI protocol and, in the case of an ambiguous result, recommends using advanced MRI techniques. When a brain MRI is inconclusive, amino acid PET can be used.

## Hyperprogression

**Fig. 7:**
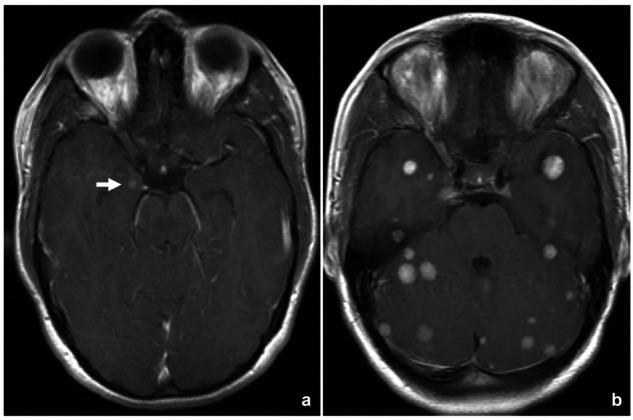
Hyperprogression in a patient with brain metastases from melanoma treated with Ipilimumab without concomitant radiotherapy. Baseline MRI scan 5 days before starting Ipilimumab (**a**) shows one 4 mm temporal lesion (arrow in **a**). Follow-up MRI scan 34 days after starting ipilimumab (**b**) demonstrates a dramatic increase in the number and size of brain metastases, with more than 5 target lesions and more than 15 non-target lesions. The SLD increased to 60 mm, and the patient also experienced a worsening of their neurological status. The rapid progression of tumor burden right after starting treatment is consistent with hyperprogression

Hyperprogression (HPD) is a phenomenon characterized by rapid acceleration of tumor growth following the initiation of immunotherapy (Fig. 7). The mechanisms underlying HPD are not well understood. A hypothesis posits that ICIs may, in some cases, promote tumor proliferation either directly through immune-mediated mechanisms such as DNA damage from free radicals or indirectly by modifying the tumor microenvironment through angiogenesis and tissue remodeling facilitated by growth factors and matrix metalloproteinases [54, 55].

A crucial limitation in the study of HPD is the variability in its definition and the criteria used for its identification. Different studies have adopted different thresholds to determine HPD. Some proposals focus on the comparison of tumor growth kinetics before and after immunotherapy [48, 56–58]. For example, HPD is characterized by a more than twofold increase in the rate of tumor volume growth after starting immunotherapy [56, 57]. Other criteria have focused on the growth of tumor volume during the first 2 months after the initiation of immunotherapy with respect to baseline volume. For instance, an increase exceeding 50% in tumor load [59], an increase surpassing 40% in tumor load, or an increase greater than 20%, accompanied by multiple new lesions [60, 61].

The absence of standardized definitions for hyperprogression (HPD) introduces significant uncertainty and controversy in the evaluation of immunotherapy response, which has led to widely varying reported incidence rates, ranging from 4% to 29% [17, 26]. Without an adequate control group, it is not possible to confirm that the acceleration of growth kinetics is induced by immunotherapy, or that similar growth kinetics simply reflect the natural history of the cancer.

Ferrara et al [48] found that approximately 14% of patients with advanced NSCLC treated with PD-1/PD-L1 inhibitors presented with HPD. Furthermore, the incidence in this group was found to be nearly three times higher than in the standard chemotherapy group, which may suggest an immune-mediated component. Additionally, patients who experienced HPD within the first 6 weeks of treatment had an overall survival that was about half that of patients with non-HPD PD.

## Dissociated response

**Fig. 8:**
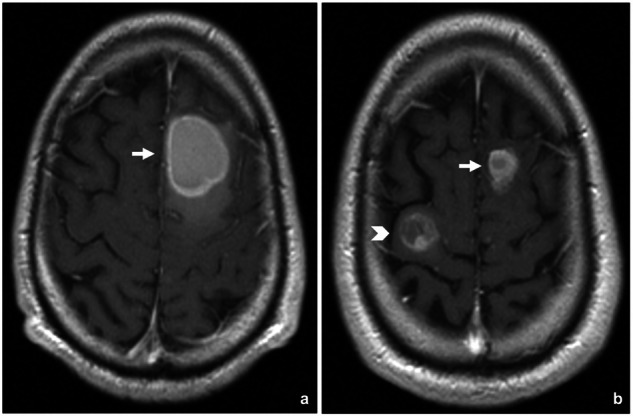
Dissociated response in a patient with brain metastases from melanoma treated with Ipilimumab and WBRT. In the baseline brain MRI (**a**), a left frontal metastasis is identified (arrow in **a**). In a subsequent follow-up scan during treatment 90 days after baseline (**b**), the initial lesion has responded to treatment, showing a significant reduction in size (arrow in **b**). However, a new lesion has appeared in the contralateral frontal lobe (arrowhead in **b**), indicating progressive disease and a dissociated response to treatment

Dissociated responses (DR) occur when some lesions grow while others regress, leading to the coexistence of both responding and non-responding metastases within the same patient (Fig. 8). There is currently no standardized definition of DR in the literature. From a pathophysiological perspective, DR is primarily attributed to tumor heterogeneity among different metastatic lesions. This heterogeneity arises from clones with varying degrees of aggressiveness [62], variable expression of immune checkpoint molecules [63], and different levels of treatment resistance. Additionally, the heterogeneity in the metastatic microenvironment, caused by tissue differences among metastatic sites, may also contribute to diverse responses [64]. It has also been proposed that treatment-secondary changes could be involved in DR (such as immune infiltration causing a transient or apparent increase in the size of some lesions) [65].

DR fundamentally challenges existing, widely used response assessment criteria like RECIST and RANO. These criteria rely on summing the diameters of target lesions to determine an overall response. A dissociated response can lead to a situation where a patient is classified as having “progressive disease” despite clear evidence of therapeutic benefit in some lesions, creating significant ambiguity in assessing treatment efficacy.

Some series indicate that DRs occur in 5–7.5% of patients with NSCLC treated with anti-PD-1/PD-L1 agents [66, 67]. Patients with DR had significantly longer overall survival than those with concordant progressive disease. To the best of our knowledge, no specific data are available regarding brain metastases treated with immunotherapy. Patients with brain metastases treated with immunotherapy exhibit response rates of approximately 45% when treated locally with extracranial radiotherapy and/or intracranial stereotactic radiosurgery for progressive lesions. Although specific recommendations for DR are lacking, it is advisable to consider local treatments for advancing lesions in these patients whenever possible, as they may improve therapeutic outcomes [68]. In this regard, DR is emerging as a favorable prognostic pattern that should be distinguished from progressive disease.

## Durable response

**Fig. 9:**
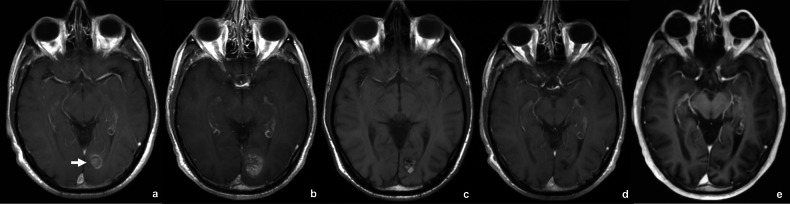
Pseudoprogression and durable response in a patient with brain metastasis from melanoma treated with Ipilimumab and WBRT. Baseline imaging, 5 days before treatment (**a**), shows a left occipital metastasis (arrow). The patient underwent four cycles of ipilimumab every 3 weeks. Follow-up imaging at 56 days after initiating ICIs (**b**) shows a 40% increase in lesion size, initially raising suspicion of PD. At 119 days post-ICI initiation (56 days after treatment discontinuation) (**c**), the lesion has shrunk by 20%, meeting the criteria for a partial response. At 182 days post-ICI initiation (118 days after treatment discontinuation) (**d**), the lesion had completely resolved, consistent with a complete response. Approximately 9 years after ICI discontinuation (**e**), no evidence of disease was detected, indicating a durable complete response. Retrospectively, the initial lesion enlargement (**b**) was identified as immunotherapy-induced pseudoprogression

The term durable response refers to a sustained response over time that can be observed even after treatment discontinuation (Fig. 9). However, an official definition is absent [26]. A recent meta-analysis defined it as progression-free survival (PFS) exceeding three times the median PFS of all patients treated with the same drugs in the same trial [69]. This study found that the proportion of patients with durable responses was 2.3 times higher in ICI arms than in non-ICI arms (25% vs. 11%).

Clinical trials have demonstrated that immunotherapy can induce durable intracranial responses in patients with asymptomatic brain metastases from melanoma, with a significant proportion maintaining long-term disease control. In these patients, achieving CR is the strongest predictor of long-term survival and minimal relapse risk after stopping therapy [70]. There is no definitive agreement regarding the optimal and safest timing for immunotherapy discontinuation in responders.

The long-term effects on immunotherapy efficacy remain unclear [71]. In patients achieving a CR or sustained clinical benefit and subsequently discontinue treatment, reintroducing ICIs upon relapse is a potential strategy (known as “re-challenge”). While some patients may respond, the likelihood of a second response is currently unknown [72].

While immunotherapy has revolutionized cancer treatment, it has also introduced significant complexity into radiological response assessment. The development of the iRECIST and iRANO criteria represented a critical step in adapting imaging evaluation to immune-related phenomena, particularly pseudoprogression. However, emerging patterns such as hyperprogression, dissociated response, and sustained response are not fully addressed within these frameworks, posing diagnostic and therapeutic challenges.

The recognition of these atypical patterns underscores several key issues. Current response criteria may not adequately capture the full spectrum of immune-related patterns, leading to the misclassification of a patient’s response or progression. Furthermore, the misinterpretation of imaging findings can result in the premature discontinuation of effective therapy or the continuation of ineffective treatment, both of which have serious implications for patient outcomes and resource utilization. Finally, inconsistent definitions and assessment standards across studies complicate trial interpretation and cross-study comparisons.

## Intracranial adverse events of immunotherapy

ICIs enhance antitumor immunity; however, they also induce several immunopathogenic mechanisms, such as cellular autoimmunity, autoantibody production, complement activation, cytokine/chemokine release, genetic factors, and gut microbiome alterations, all of which are implicated in the development of immune-related adverse events (irAEs) [73].

Gastrointestinal, dermatologic, hepatic, endocrine, and pulmonary toxicities are the most common irAEs, some of which can lead to emergency department visits in these patients [73]. Intracranial irAEs are infrequent, but they may occur in patients with brain metastases, complicating differential diagnosis. irAEs are more commonly observed with anti-CTLA-4 agents than with anti-PD-1/PD-L1 therapies [74], with the highest incidence noted in combination regimens [75]. These effects typically manifest within the first months of treatment, although they can also occur following a single dose or even years later [76]. The ASCO-NCCN guidelines [77] stipulate that the management of irAEs is based on severity: grade 1 involves continuous monitoring, grade 2 usually requires withholding ICI and considering corticosteroids, grade 3 necessitates high-dose corticosteroids ± immunosuppression, and grade 4 mandates permanent discontinuation. This review focuses on the most frequent intracranial irAEs, specifically hypophysitis and encephalitis.

## Hypophysitis

**Fig. 10:**
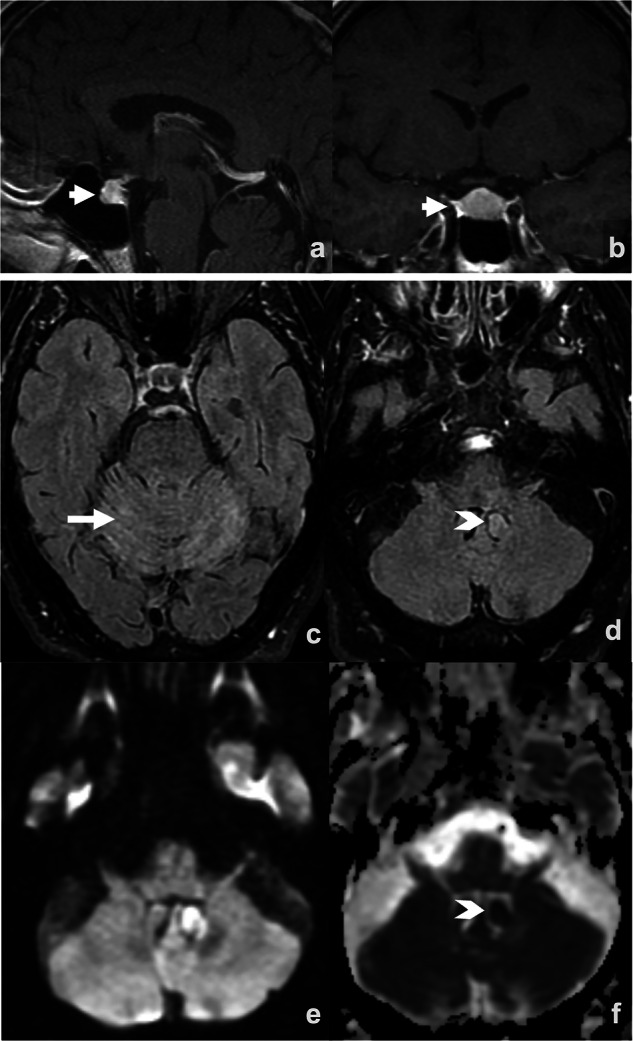
Intracranial immune-related adverse events. Ipilimumab-induced hypophysitis in a patient with brain metastases from melanoma, without concomitant radiotherapy (**a**, **b**). Two months after starting immunotherapy, the patient developed panhypopituitarism. An MRI revealed an enlarged, enhancing pituitary gland (arrow in **a** and **b**). Pembrolizumab-induced cerebellitis in a patient with brain metastases from melanoma without concomitant radiotherapy (**c**–**f**). Five days after treatment began, the patient developed ataxia and dysmetria. An MRI showed T2/FLAIR and diffusion hyperintensity in the cerebellar parenchyma (arrow in **c**) and left tonsil (arrowhead in **d**); ADC maps showed cytotoxic edema (arrowhead in **f**). CSF analysis was positive for anti-GluK2 antibodies

Endocrine irAEs of clinical significance occur in approximately 10% of patients treated with ICIs [78]. While thyroid disorders are the most common endocrine irAE in patients receiving anti-PD(L)1-based therapies and are rarely severe; hypophysitis mostly occurs in patients treated with ipilimumab, and early recognition is crucial due to its life-threatening nature [79]. This condition is characterized by pituitary inflammation leading to hormonal dysfunction. Clinical manifestations encompass fatigue, headache, visual disturbances, hypotension, and hyponatremia. Contrast-enhanced brain MRI is the imaging modality of choice for the evaluation of hypophysitis, as it depicts early pituitary enhancement and glandular enlargement (Fig. 10), often preceding the onset of clinical symptoms [80]. Management includes hormonal replacement and temporary ICI discontinuation. In contrast to other irAEs, endocrine toxicities generally allow ICI reintroduction once the patient is stabilized. Pituitary function recovery is variable, and many patients require long-term hormonal therapy.

## Encephalitis and other irAEs in the CNS

Neurological irAEs are rare, occurring in approximately 1% of cases [81], with neuromuscular syndromes being the most prevalent. Central nervous system involvement is even less frequent, manifesting a broad clinical spectrum that ranges from headache to severe syndromes, such as meningitis, encephalitis, transverse myelitis, or posterior reversible encephalopathy syndrome. Diagnosis relies on brain MRI and cerebrospinal fluid analysis. Encephalitis is the most prevalent subtype (13% of neurological irAEs) [82], typically presenting with headache, altered mental status, and focal deficits. Workup should rule out tumor progression, infection, metabolic disturbances, and paraneoplastic syndromes. Brain MRI, although sometimes normal, may reveal T2/FLAIR or diffusion abnormalities (Fig. 10).

Less common intracranial irAEs include aseptic meningitis, transverse myelitis, and cranial nerve involvement [81]. Aseptic meningitis typically presents with headache, photophobia, and nuchal rigidity, and may show leptomeningeal enhancement on MRI [83]. Transverse myelitis is a severe irAE characterized by bilateral sensory and/or motor deficits, often with hyperreflexia. Diagnosis requires high-resolution spinal MRI with thin-slice axial sections, along with brain MRI, both with and without contrast. ICI discontinuation is advised in all cases [77].

## Hemorrhagic transformation of brain metastases

**Fig. 11:**
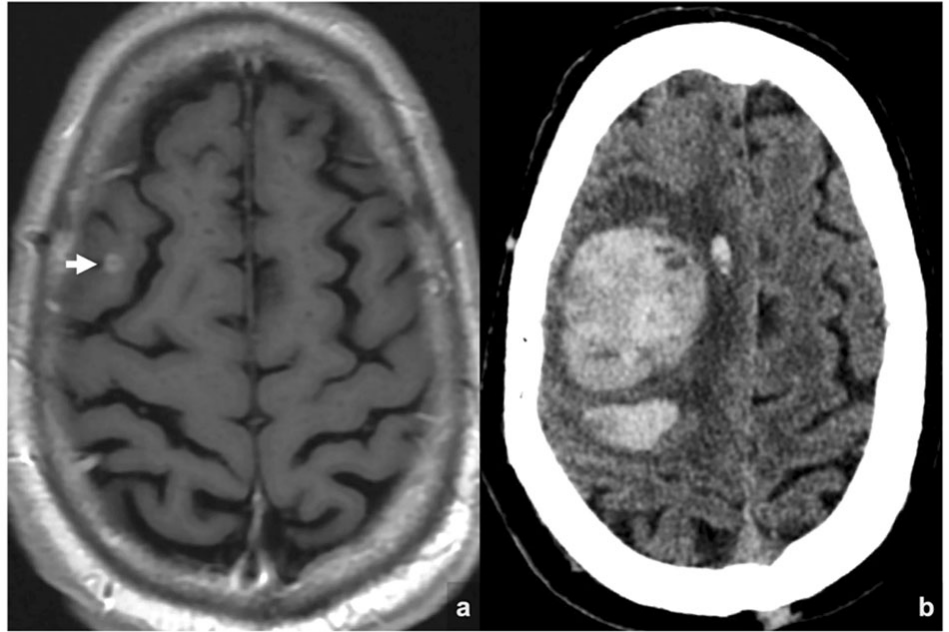
Hemorrhagic transformation of metastasis from melanoma treated with Nivolumab–Ipilimumab and WBRT. Brain MRI performed 51 days prior to the initiation of treatment (**a**) showed a right frontal metastasis. Fifty-two days after starting treatment, the patient presented to the emergency department with sudden-onset left hemiparesis. A cranial CT scan (**b**) performed the same day revealed a right frontal intraparenchymal hemorrhage consistent with hemorrhagic transformation of the known brain metastasis

Some studies have reported an increased incidence of intratumoral hemorrhage in melanoma brain metastases treated with a combination of ipilimumab and WBRT [84, 85] (Fig. 11). However, other studies found no significant differences in hemorrhagic events when ipilimumab was added to WBRT compared to WBRT alone [86]. While existing evidence does not confirm an increased risk of hemorrhage with this combination, it highlights the need for prospective studies aimed at specifically evaluating intratumoral bleeding.

## Posterior reversible encephalopathy syndrome

**Fig. 12:**
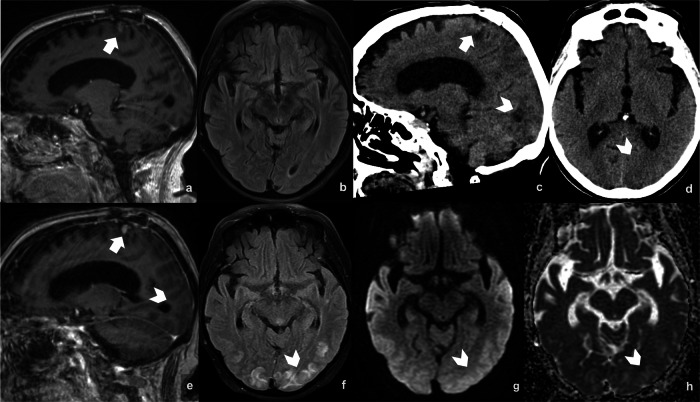
PRES in a patient with NSCLC brain metastasis treated with Pembrolizumab and SRS. Baseline MRI (**a**, **b**) shows recurrence of a left precentral metastasis (arrow). Eleven days after treatment, the patient presented with hypertension and altered mental status. CT (**c**, **d**) revealed bilateral parieto-occipital hypodensities (arrowheads), suggestive of PRES. MRI (**e**–**h**) confirmed bilateral parieto-occipital T1 hypointense areas (**e**), T2-FLAIR hyperintense regions (**f**), and facilitated diffusion on DWI/ADC (**g**, **h**), consistent with a PRES diagnosis

Posterior reversible encephalopathy syndrome (PRES) is characterized by subcortical vasogenic brain edema, predominantly in the parieto-occipital regions (Fig. 12). Neuroimaging typically reveals white matter edema and may display hemorrhage, restricted diffusion, or contrast enhancement [87]. In most cases, it is reversible and has a favorable prognosis. It has been associated with various conditions, including renal failure, cytotoxic drugs, or autoimmune disorders [87]. Some case reports have described the occurrence of PRES following the initiation of immunotherapy [88]. However, no series has been published to establish a definitive association.

## Conclusion

Radiologists must be well-versed in both classical and atypical response patterns to immunotherapy in brain metastases, along with potential adverse events. Atypical response patterns, including pseudoprogression, dissociated response, hyperprogression, and intracranial immune-mediated adverse events, such as encephalitis, might make the assessment of therapy response more complex. Brain MRI is an essential tool for evaluating response, whereas advanced imaging techniques, such as perfusion MRI, MRS, and amino acid PET, serve as complementary techniques to differentiate between progression and post-treatment changes. This allows a more accurate evaluation to guide appropriate treatment decisions and enhance patient outcomes.

## Data Availability

The materials analyzed during the current work are available from the corresponding author on reasonable request.

## Abbreviations

BBB: Blood-brain barrier
CBV: Cerebral blood volume
Cho: Choline
CR: Complete response
Cr: Creatine
CT: Computed tomography
CTLA-4: Cytotoxic T-lymphocyte-associated protein 4
DCE: Dynamic contrast-enhanced
DR: Dissociated responses
DSC: Dynamic susceptibility contrast
DWI: Diffusion-weighted imaging
FLAIR: Fluid-attenuated inversion recovery
HPD: Hyperprogression
ICIs: Immune checkpoint inhibitors
irAEs: Immune-related adverse events
Ktrans: Leakage constant
Lac: Lactate
Lip: Lipids
mRECIST: Modified RECIST
MRI: Magnetic resonance imaging
MRS: Magnetic resonance spectroscopy
NAA: N-acetyl aspartate
NCCN: National Comprehensive Cancer Network
NSCLC: Non-small-cell lung cancer
PD: Progressive disease
PD-1: Programmed cell death protein 1
PDL-1: Programmed death-ligand 1
PET: Positron emission tomography
PFS: Progression-free survival
PR: Partial response
PRES: Posterior reversible encephalopathy syndrome
PsPD: Pseudoprogression
RANO: Response assessment in neuro-oncology
RANO-BM: Response assessment in neuro-oncology-brain metastases
RANO-HGG: Response assessment in neuro-oncology high-grade gliomas
RECIST: Response evaluation criteria in solid tumors
RN: Radiation necrosis
SD: Stable disease
SLD: Sum of longest diameters
SRS: Stereotactic radiosurgery
WBRT: Whole-brain radiotherapy
